# Meditating on Cancer Management at the Time of Immunotherapy

**DOI:** 10.3390/jcm11113025

**Published:** 2022-05-27

**Authors:** Egesta Lopci

**Affiliations:** Nuclear Medicine, IRCCS—Humanitas Research Center, Via Manzoni 56, 20089 Rozzano, MI, Italy; egesta.lopci@gmail.com or egesta.lopci@cancercenter.humanitas.it

The introduction of checkpoint inhibitors in the last decade has prompted a new era in medical oncology and has opened the door to novel frontiers in cancer treatment. Immunotherapy has determined unprecedented results in various cancer types, but also new challenges and opportunities in patient management. From an imaging point of view, the difficulties encountered in tumor response assessment have been a major problem for radiologists and nuclear medicine physicians, leading, along the years, to continuous adaptations of available response criteria [1,2]. In this context, the recent joint guidelines/procedure standards prepared as collaboration between the European Association of Nuclear Medicine (EANM), the Society of Nuclear Medicine and Molecular Imaging (SNMMI), and the Australian and New Zealand Society of Nuclear Medicine (ANZSNM) on the recommended use of [^18^F]FDG PET/CT in cancer patients candidate to immunomodulatory drugs can become a cornerstone for metabolic response assessment during immunotherapy with checkpoint inhibitors [3]. To be able to overcome challenges and maximize treatment efficacy, it is important to know current standards of cancer management and get insight into perspectives of clinical research at the time of immunotherapy. These represent the aims of this Special Issue in the *Journal of Clinical Medicine*, focusing on “Cancer Management in the Era of Immunotherapy” and presenting a series of nine papers (four original articles and five reviews) submitted by international leaders in the field. 

Linuma and colleagues [4] open the issue by discussing the prognostic and predictive role of neutrophil-to-lymphocyte ratio (NLR), the platelet-to-lymphocyte ratio (PLR), and systemic immune inflammation (SII) indexes in patients with metastatic renal cell carcinoma (MRCC) treated with combination immunotherapy, i.e., nivolumab and ipilimumab. All abovementioned parameters were found able to differentiate patients having a higher rate of 1-year progression-free survival (PFS) from those having a poorer outcome, thus confirming the promising results obtained in similar contexts with other tumor types investigated, also with [^18^F]FDG PET/CT [5,6,7,8]. In fact, the combination of metabolic information on PET with other clinical and laboratory data can provide useful information in various therapeutic regimens, including checkpoint inhibitors, particularly in tumors such as melanomas and non-small cell lung cancer (NSCLC) [7]. To better cover the argument, two dedicated reviews [9,10] and two original articles [9,10] have been provided describing the impact of [^18^F]FDG PET/CT at different timing in both abovementioned tumors. Liberini and colleagues [11] investigated patients with advanced melanoma undergoing target therapy or immunotherapy and correlated baseline information on PET with tumor response, PFS and OS, proving that poorer outcome was correlated to higher values of whole-body and bone metabolic parameters, whereas OS was correlated to higher values of whole-body, lymph node, and soft tissue metabolic parameters. In patients with NSCLC, Castello and colleagues [12] assessed instead the role of antibiotic therapy (ATB) effect of immune checkpoint inhibitors (ICI) efficacy. By comparing results in patients receiving ATB within 1 month from the first dose of ICI, the authors documented a worse response to therapy and a poorer PFS in those patients treated with ATB, together with those having a performance status ECOG ≥ 1, and a lower ∆SUVmax (<−16.9). These results should not be surprising, as it is largely renowned that gut microbiota directly influences the therapeutic efficacy of ICI [13], while antibiotics negatively impact on the quality of the microbiota. This argument has also been discussed in the review article prepared by Tran and colleagues [14], who focused particularly on the effect of ICI and microbiota in the occurrence of gastrointestinal immune-related adverse events (irAEs), whereas a dedicated review on cutaneous irAES, illustrating their clinical and histopathological features as well as outcome, has been presented by Hashimoto and colleagues [15]. 

Thanks to the contributions provided by Franzi and colleagues [16] and Knetki-Wróblewska and colleagues [15], the Special Issue has been enriched with two review articles facing the potentials of chemo-immunotherapy in locally advanced NSCLC patients [16] and the therapeutic opportunities in those cases with coexistence of high PD-L1 expression and RET fusion [17], respectively. Clearly, the debate on the best treatment options in the era checkpoint inhibitors is far from being over; nevertheless, the literature reviews and insights provided by the abovementioned authors can help an attentive reader to shed some light on the arguments. 

In the perspectives of optimal patients selection and outcome prediction, special attention must be given to the role of artificial intelligence and radiomics. With regard to imaging, much progress has been made, but also much more remains to be achieved. To help better understand the topic, a dedicated article has been prepared by Castello and colleagues [10], providing a comprehensive review on radiomics in the era of ICI as a new protagonist in the “jungle” of response criteria. By means of this article [10], together with the last contribution [9], the Special Issue has been complemented with up-to-date knowledge on advanced metabolic imaging in cancer patients candidate to immunotherapy.

## Data Availability

Not applicable.

## References

[1] Rossi S., Toschi L., Castello A., Grizzi F., Mansi L., Lopci E. (2017). Clinical characteristics of patient selection and imaging predictors of outcome in solid tumors treated with checkpoint-inhibitors. Eur. J. Nucl. Med. Mol. Imaging.

[2] Aide N., Hicks R.J., Le Tourneau C., Lheureux S., Fanti S., Lopci E. (2019). FDG PET/CT for assessing tumour response to immunotherapy: Report on the EANM symposium on immune modulation and recent review of the literature. Eur. J. Nucl. Med. Mol. Imaging.

[3] Lopci E., Hicks R.J., Dimitrakopoulou-Strauss A., Dercle L., Iravani A., Seban R.D., Sachpekidis C., Humbert O., Gheysens O., Glaudemans A.W.J.M. (2022). Joint EANM/SNMMI/ANZSNM practice guidelines/procedure standards on recommended use of [18F]FDG PET/CT imaging during immunomodulatory treatments in patients with solid tumors version 1.0. Eur. J. Nucl. Med. Mol. Imaging.

[4] Iinuma K., Enomoto T., Kawada K., Fujimoto S., Ishida T., Takagi K., Nagai S., Ito H., Kawase M., Nakai C. (2021). Utility of Neutrophil-to-Lymphocyte Ratio, Platelet-to-Lymphocyte Ratio, and Systemic Immune Inflammation Index as Prognostic, Predictive Biomarkers in Patients with Metastatic Renal Cell Carcinoma Treated with Nivolumab and Ipilimumab. J. Clin. Med..

[5] Castello A., Toschi L., Rossi S., Mazziotti E., Lopci E. (2020). The immune-metabolic-prognostic index and clinical outcomes in patients with non-small cell lung carcinoma under checkpoint inhibitors. J. Cancer Res. Clin. Oncol..

[6] Castello A., Rossi S., Mazziotti E., Toschi L., Lopci E. (2020). Hyperprogressive Disease in Patients with Non-Small Cell Lung Cancer Treated with Checkpoint Inhibitors: The Role of 18F-FDG PET/CT. J. Nucl. Med..

[7] Seban R.D., Moya-Plana A., Antonios L., Yeh R., Marabelle A., Deutsch E., Schwartz L.H., Gómez R.G.H., Saenger Y., Robert C. (2020). Prognostic 18F-FDG PET biomarkers in metastatic mucosal and cutaneous melanoma treated with immune checkpoint inhibitors targeting PD-1 and CTLA-4. Eur. J. Nucl. Med. Mol. Imaging.

[8] Bauckneht M., Genova C., Rossi G., Rijavec E., Dal Bello M.G., Ferrarazzo G., Tagliamento M., Donegani M.I., Biello F., Chiola S. (2021). The Role of the Immune Metabolic Prognostic Index in Patients with Non-Small Cell Lung Cancer (NSCLC) in Radiological Progression during Treatment with Nivolumab. Cancers.

[9] Lopci E. (2021). Immunotherapy Monitoring with Immune Checkpoint Inhibitors Based on [18F]FDG PET/CT in Metastatic Melanomas and Lung Cancer. J. Clin. Med..

[10] Castello A., Castellani M., Florimonte L., Urso L., Mansi L., Lopci E. (2022). The Role of Radiomics in the Era of Immune Checkpoint Inhibitors: A New Protagonist in the Jungle of Response Criteria. J. Clin. Med..

[11] Liberini V., Rubatto M., Mimmo R., Passera R., Ceci F., Fava P., Tonella L., Polverari G., Lesca A., Bellò M. (2021). Predictive Value of Baseline [18F]FDG PET/CT for Response to Systemic Therapy in Patients with Advanced Melanoma. J. Clin. Med..

[12] Castello A., Rossi S., Toschi L., Lopci E. (2021). Impact of Antibiotic Therapy and Metabolic Parameters in Non-Small Cell Lung Cancer Patients Receiving Checkpoint Inhibitors. J. Clin. Med..

[13] Patel P., Poudel A., Kafle S., Thapa Magar M., Cancarevic I. (2021). Influence of Microbiome and Antibiotics on the Efficacy of Immune Checkpoint Inhibitors. Cureus.

[14] Tran T., Tran N.G.T., Ho V. (2022). Checkpoint Inhibitors and the Gut. J. Clin. Med..

[15] Hashimoto H., Ito T., Ichiki T., Yamada Y., Oda Y., Furue M. (2021). The Clinical and Histopathological Features of Cutaneous Immune-Related Adverse Events and Their Outcomes. J. Clin. Med..

[16] Franzi S., Mattioni G., Rijavec E., Croci G.A., Tosi D. (2022). Neoadjuvant Chemo-Immunotherapy for Locally Advanced Non-Small-Cell Lung Cancer: A Review of the Literature. J. Clin. Med..

[17] Knetki-Wróblewska M., Wojas-Krawczyk K., Kowalski D.M., Krzakowski M. (2022). Non-Small-Cell Lung Cancer Patients with Coexistence of High PD-L1 Expression and RET Fusion—Which Path Should We Follow? Case Reports and Literature Review. J. Clin. Med..

